# Supplementary material for: Genome-Wide Association Analysis Identifies Candidate Loci for Callus Induction in Rice (Oryza sativa L.)
Source: Plants (Basel). 2024 Jul 30;13(15):2112. doi: 10.3390/plants13152112 (PMC11314294; doi:10.3390/plants13152112)
Supplement: Supplementary file 1 [file plants-13-02112-s001.zip › plants-3105989-supplementary.pdf]

Supplementary Materials

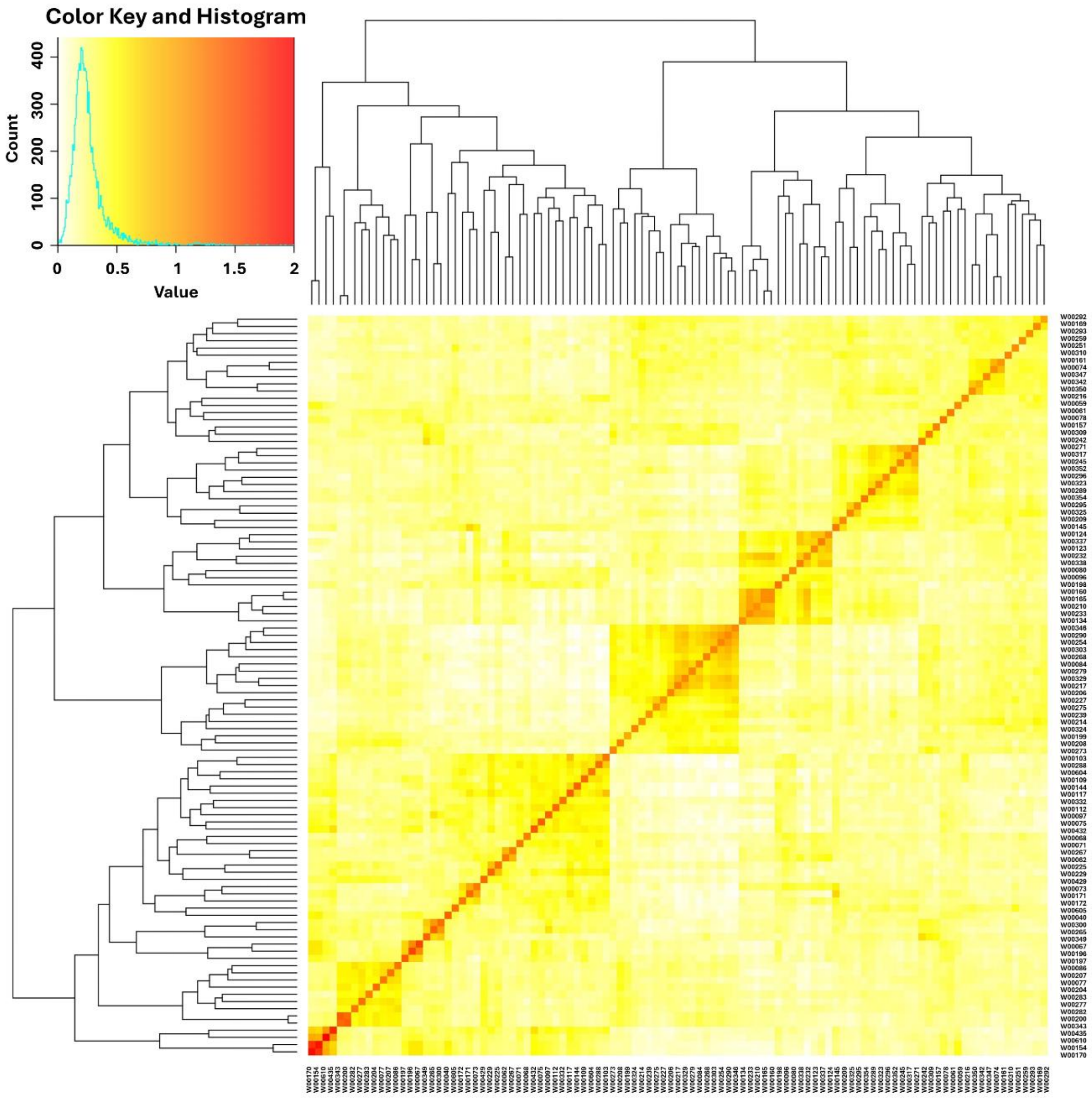

Supplementary Figure S1 shows the kinship matrix of the 110 individuals displayed as a heatmap.

Supplementary Table S1 A list of 289 rice accessions and their seed germination percentage in the MS media plus 3% sucrose

Item	seed code	GWAS code	Name	Nubmer of seed test	Number of germinate seed	% Germination
1	WS21-S5-001	DP027	Sai Rai	10	8	80
2	WS21-S5-003	GW071	T6-4	10	4	40
3	WS21-S5-004	GW141	RD9	10	9	90
4	WS21-S5-005	DP032	Puang Nak	10	7	70
5	WS21-S5-006	W00003	Mu9962	10	6	60
6	WS21-S5-007	GW060	Mudgo	10	9	90
7	WS21-S5-008	GW195	Plong Awe	10	9	90
8	WS21-S5-009	GW129	RD43	10	6	60
9	WS21-S5-010	GW176	Dor Samdeun	10	9	90
10	WS21-S5-011	GW044	IR58	10	5	50
11	WS21-S5-012	W00115	DHARIA	10	7	70
12	WS21-S5-013	DP011	Kao Prapass	10	4	40
13	WS21-S5-014	GW186	Num Sagui 19	10	10	100
14	WS21-S5-015		Kao Prakarn	10	5	50
15	WS21-S5-016	DP035	Rak Hang	10	10	100
16	WS21-S5-017	GW038	IR29	10	2	20
17	WS21-S5-018	DP031	Puang Sunng	10	6	60
18	WS21-S5-019	W00001	Jao Homnin	10	7	70
19	WS21-S5-020	GW243	Hom PooKaew	10	10	100
20	WS21-S5-021	GW261	Hom Baorai	10	7	70
21	WS21-S5-024	DP019	Dor Dokmai	10	5	50
22	WS21-S5-025	W00040	Pokkali	10	10	100
23	WS21-S5-026	GW024	FL530	10	9	90
24	WS21-S5-027	GW132	RD49	10	2	20
25	WS21-S5-028	GW009	BRI1-13-B-55	10	6	60
26	WS21-S5-029	GW104	Phae	10	7	70
27	WS21-S5-030	GW008	BENARA	10	10	100
28	WS21-S5-031	DP022	Tom Muangluang	10	10	100
29	WS21-S5-032	GW132	RD49 (RD)	10	7	70
30	WS21-S5-033	GW018	CT9993	10	4	40
31	WS21-S5-034	GW237	Hom Keekway	10	5	50
32	WS21-S5-035	GW115	RD15	10	7	70
33	WS21-S5-036	GW037	IR1188	10	5	50
34	WS21-S5-037		Dok Kao	10	9	90
35	WS21-S5-038	GW187	Bug Muai	10	9	90
36	WS21-S5-039	GW180	Tub Meidum	10	9	90
37	WS21-S5-040	W00597	DV85	10	4	40
38	WS21-S5-041	GW045	IR60	10	4	40
39	WS21-S5-042	GW229	Suphan Buri 60	10	7	70
40	WS21-S5-043	GW165	Kee Tomphan	10	9	90
41	WS21-S5-044	GW173	Dor Mei	10	7	70
42	WS21-S5-045	DP033	Muang Nga	10	7	70
43	WS21-S5-046	GW023	FL496	10	9	90
44	WS21-S5-047	GW135	RD57	10	9	90

Item	seed code	GWAS code	Name	Nubmer of seed test	Number of germinate seed	% Germination
45	WS21-S5-048	GW081	Jao Kao Chiang Mai	10	7	70
46	WS21-S5-049	GW006	BA7	10	10	100
47	WS21-S5-050	GW005	Azucena	10	10	100
48	WS21-S5-051	GW106	Kai Noiluang	10	6	60
49	WS21-S5-052	GW252	Kao Dok Mali 105	10	7	70
50	WS21-S5-053	GW047	IR64	10	8	80
51	WS21-S5-054	GW244	Hom Lao	10	10	100
52	WS21-S5-055	GW174	Dok Phayom	10	10	100
53	WS21-S5-056	GW209	Phisanulok 2	10	7	70
54	WS21-S5-057	GW103	Kan Chan	10	10	100
55	WS21-S5-058	GW061	Nanoboka	10	7	70
56	WS21-S5-059	DP017	Dok Ka	10	8	80
57	WS21-S5-060	GW149	Kao Jao Hom Suphan Buri	10	10	100
58	WS21-S5-061	GW036	Sinthulette-Saltol	10	8	80
59	WS21-S5-062	DP032	Phuang Hangnak	10	6	60
60	WS21-S5-063	GW250	Epid	10	10	100
61	WS21-S5-064	DP036	Lao Taak	10	2	20
62	WS21-S5-065	GW028	HomMali803-2	10	10	100
63	WS21-S5-066	GW026	HomMali802	10	7	70
64	WS21-S5-067	DP030	Hang Nak	10	5	50
65	WS21-S5-068	GW016	CSSL-Chr1(16)	10	8	80
66	WS21-S5-069	GW102	Luang Hom	10	6	60
67	WS21-S5-070	GW251	Niaw Hom (Niaw)	10	9	90
68	WS21-S5-071	GW017	CSSL-Chr8(106)	10	3	30
69	WS21-S5-072	GW164	Kee Tom Kamnun	10	8	80
70	WS21-S5-073	GW099	Luang Kamin	10	10	100
71	WS21-S5-074	GW050	Jasmine IR57514(BC4F7)	10	3	30
72	WS21-S5-075	GW119	RD23	10	9	90
73	WS21-S5-076	R3	Jasmine85	10	9	90
74	WS21-S5-077	GW171	Chai Nart 2	10	4	40
75	WS21-S5-078	GW090	Niaw Dum	10	10	100
76	WS21-S5-079	GW048	IR72	10	7	70
77	WS21-S5-080	GW121	RD29 (Chai Nart 80)	10	3	30
78	WS21-S5-081	GW063	NO.51-PSL	10	3	30
79	WS21-S5-082	GW170	Chai Nart 1	10	9	90
80	WS21-S5-083	GW227	Suphan Buri 2	10	10	100
81	WS21-S5-084		CSSL-Chr(II)	10	3	30
82	WS21-S5-085	GW245	Hom Suphan	10	9	90
83	WS21-S5-086	GW004	Abhaya	10	5	50
84	WS21-S5-087	GW123	RD33 (Hom Ubon 80)	10	8	80
85	WS21-S5-088	GW136	RD6	10	10	100
86	WS21-S5-089		RD6 lab	10	8	80
87	WS21-S5-090	GW031	HomMali 821	10	5	50

Item	seed code	GWAS code	Name	Nubmer of seed test	Number of germinate seed	% Germination
88	WS21-S5-091	GW216	Mun Phed 215-33-65	10	8	80
89	WS21-S5-093	GW095	Niaw Ubon 2	10	9	90
90	WS21-S5-094	GW109	Ai Tai	10	9	90
91	WS21-S5-095	GW172	Chum Pae 60	10	10	100
92	WS21-S5-096	GW072	T6-6	10	10	100
93	WS21-S5-097	GW065	PK1-PY-BLB-2	10	7	70
94	WS21-S5-098	GW225	Sin Lak	10	9	90
95	WS21-S5-099	GW282	Hom Lanna	10	5	50
96	WS21-S5-100	GW096	Mei Nong 62M	10	7	70
97	WS21-S5-101	DP018	Dok Mai	10	8	80
98	WS21-S5-102	GW001	1094-61	10	5	50
99	WS21-S5-103	DP004	Kalubala Vee	10	10	100
100	WS21-S5-104	GW142	RD-Maejo 2	10	6	60
101	WS21-S5-105	GW041	IR49830	10	7	70
102	WS21-S5-106	GW111	RD10	10	7	70
103	WS21-S5-107	GW085	Chiang Dao	10	3	30
104	WS21-S5-108	GW097	Luang Yai	10	3	30
105	WS21-S5-109		KDML	10	6	60
106	WS21-S5-110	GW101	Luang Plala	10	4	40
107	WS21-S5-111		HomMali822	10	6	60
108	WS21-S5-112	GW181	Thunyasirin	10	9	90
109	WS21-S5-113	DP039	Hom Nangnual	10	10	100
110	WS21-S5-114	DP045	Luang Chum Pae	10	9	90
111	WS21-S5-115	GW228	Suphan Buri 3	10	6	60
112	WS21-S5-116	GW003	71654R	10	7	70
113	WS21-S5-117	GW118	RD21	10	5	50
114	WS21-S5-118	GW128	RD41	10	9	90
115	WS21-S5-119	GW239	Hom Chonlasit	10	5	50
116	WS21-S5-120		TS2	10	10	100
117	WS21-S5-121	GW114	RD14	10	8	80
118	WS21-S5-122	GW188	Nang Taan	10	3	30
119	WS21-S5-123	GW092	Niaw Sanpatong	10	10	100
120	WS21-S5-124	DP005	RD53	10	9	90
121	WS21-S5-125	W00435	DH103	10	10	100
122	WS21-S5-126	GW177	Dee Ngoolaum	10	4	40
123	WS21-S5-127	GW235	Hom Dangnoi	10	2	20
124	WS21-S5-128		Kao Makkaak	10	5	50
125	WS21-S5-129	GW075	TDK1-Sub	10	10	100
126	WS21-S5-130	GW079	Kiaw Leu	10	5	50
127	WS21-S5-131		Kao Hom	10	3	30
128	WS21-S5-132	R8	RD4	10	10	100
129	WS21-S5-133	W00016	PTT1	10	7	70
130	WS21-S5-134	GW054	KDML105-Bph3-1	10	10	100
131	WS21-S5-135	GW093	Kiaw Hom	10	7	70
132	WS21-S5-136	GW035	HomMali841	10	3	30
133	WS21-S5-137	DP024	Ta Kiad	10	9	90
134	WS21-S5-138	GW030	HomMali805	10	5	50

Item	seed code	GWAS code	Name	Nubmer of seed test	Number of germinate seed	% Germination
135	WS21-S5-139	GW241	Hom Naiphon	10	9	90
136	WS21-S5-140	GW148	Kao Jao Hom Phissanulok	10	5	50
137	WS21-S5-141	GW040	IR4563-52-1-3-6	10	8	80
138	WS21-S5-142	GW215	Mali Gomain	10	9	90
139	WS21-S5-143	GW192	Pathum Thani 60	10	9	90
140	WS21-S5-144	GW197	Pin Kaset 1	10	9	90
141	WS21-S5-145		TDK1	10	10	100
142	WS21-S5-146	GW134	RD51 Hom Mali 801	10	7	70
143	WS21-S5-147	GW025	FR13A	10	6	60
144	WS21-S5-148	GW094	Niaw Ubon 1	10	10	100
145	WS21-S5-149	DP023	Tapao Lom	10	4	40
146	WS21-S5-150	GW067	PSL85063-9-1-2	10	9	90
147	WS21-S5-151	GW027	HomMali803-1	10	10	100
148	WS21-S5-152	GW033	HomMali823	10	5	50
149	WS21-S5-153	GW014	CNTLR82006-KSR-2-7	10	10	100
150	WS21-S5-154		Phuang Kaaw	10	8	80
151	WS21-S5-155	DP025	Ta Ban	10	10	100
152	WS21-S5-156	DP034	Ruang Diaw	10	10	100
153	WS21-S5-157	GW140	RD8	10	10	100
154	WS21-S5-158	GW122	RD31 (Pathum Thani 80)	10	9	90
155	WS21-S5-159	GW125	RD37	10	10	100
156	WS21-S5-161		SPRLR79077-KSR-2-2	10	7	70
157	WS21-S5-162	GW184	Nangmol S4	10	9	90
158	WS21-S5-163	W00432	IR62266	10	10	100
159	WS21-S5-164		HomMali831	10	10	100
160	WS21-S5-165	GW230	Suphan Buri 90	10	10	100
161	WS21-S5-166	GW124	RD35 (Rangsit 80)	10	8	80
162	WS21-S5-167	DP040	Hom Sutabud	10	10	100
163	WS21-S5-168	GW139	RD7	10	10	100
164	WS21-S5-169	GW074	TDK1_Aroma	10	8	80
165	WS21-S5-170	R7	RD39	10	10	100
166	WS21-S5-171	GW147	Kao Jao Hom Klong Lueng 1	10	10	100
167	WS21-S5-172	GW130	RD45	10	8	80
168	WS21-S5-173	GW271	IR40	10	10	100
169	WS21-S5-174	W00429	MNTK75	10	10	100
170	WS21-S5-175	W00064	RD47	10	10	100
171	WS21-S5-176	GW154	Kao Kordiaw 35	10	10	100
172	WS21-S5-177	GW013	CNTBR82040-259-1-1-1	10	10	100
173	WS21-S5-178	GW224	Sun Patong 1	10	7	70
174	WS21-S5-179	GW231	SPR4	10	10	100
175	WS21-S5-180	GW226	Suphan Buri 1	10	10	100
176	WS21-S5-181	DP014	Jao Hom	10	7	70

Item	seed code	GWAS code	Name	Nubmer of seed test	Number of germinate seed	% Germination
177	WS21-S5-182	GW233	Maknum	10	10	100
178	WS21-S5-183	GW203	Pamar Dum	10	10	100
179	WS21-S5-184	GW055	KDML105-PlusIII	10	9	90
180	WS21-S5-185	GW112	RD11	10	10	100
181	WS21-S5-186	W00611	RD5	10	9	90
182	WS21-S5-188	GW116	RD17	10	3	30
183	WS21-S5-189	GW043	IR57514	10	4	40
184	WS21-S5-190	GW020	DH212	10	7	70
185	WS21-S5-191	GW053	KD20	10	10	100
186	WS21-S5-192	GW240	Hom Dong	10	10	100
187	WS21-S5-193	GW242	Hom Pamar	10	10	100
188	WS21-S5-194	DP012	Koo Muang	10	9	90
189	WS21-S5-195	DP007	Kao Kum	10	10	100
190	WS21-S5-196	GW042	IR53936	10	7	70
191	WS21-S5-197	GW105	Mae Lad	10	10	100
192	WS21-S5-198		IWSIO	10	7	70
193	WS21-S5-199	GW163	Kee Tomklang	10	10	100
194	WS21-S5-200	GW146	Koo Muang Lueng	10	9	90
195	WS21-S5-201	DP046	Leung Dong	10	10	100
196	WS21-S5-202	GW120	RD27	10	10	100
197	WS21-S5-203	GW213	Pissanulok 80	10	10	100
198	WS21-S5-204	GW232	Surin 1	10	10	100
199	WS21-S5-205	GW155	Kao Nagchang	10	10	100
200	WS21-S5-206	GW183	Nang Chalong	10	4	40
201	WS21-S5-207	GW162	Kao Panlueng	10	8	80
202	WS21-S5-208	GW086	Lebnok Pattani	10	7	70
203	WS21-S5-209	GW108	Riceberry	10	10	100
204	WS21-S5-210	GW056	Knga Kyein Thee	10	9	90
205	WS21-S5-211	GW221	See Nual	10	10	100
206	WS21-S5-212	DP001	Dular	10	10	100
207	WS21-S5-213	GW214	Mali Dang	10	10	100
208	WS21-S5-214	GW097	Luang Yai	10	10	100
209	WS21-S5-215	DP016	Chor Makok	10	10	100
210	WS21-S5-216	GW057	MeeKauk	10	10	100
211	WS21-S5-217	GW252	Kao Dok Mali	10	8	80
212	WS21-S5-218	DP047	Luang Tong	10	10	100
213	WS21-S5-219	GW152	Kao Konjud	10	9	90
214	WS21-S5-220	GW248	Harruang Bao	10	7	70
215	WS21-S5-221	GW198	Pinkaset 3	10	10	100
216	WS21-S5-222	DP044	Luang Ngam	10	8	80
217	WS21-S5-223	GW211	Pissanulok 60-1	10	10	100
218	WS21-S5-224	DP038	Hom Tong	10	10	100
219	WS21-S5-225	GW217	Mar Bung	10	10	100
220	WS21-S5-226	GW199	Pinkaset 4	10	10	100
221	WS21-S5-227	GW158	Kao Nok	10	10	100
222	WS21-S5-229	GW168	Chumpa Son	10	8	80
223	WS21-S5-230	GW153	Kao Klang	10	10	100

Item	seed code	GWAS code	Name	Nubmer of seed test	Number of germinate seed	% Germination
224	WS21-S5-231	DP026	Tong Raksai	10	10	100
225	WS21-S5-232	GW210	Pissanulok 3	10	8	80
226	WS21-S5-234	GW151	Kao Pongkai	10	10	100
227	WS21-S5-235	GW206	Puang Nak	10	10	100
228	WS21-S5-236	GW138	RD69 (Tabtim Chumpae)	10	5	50
229	WS21-S5-237	GW222	Sung Yod	10	10	100
230	WS21-S5-238	GW189	Beu Sormee	10	9	90
231	WS21-S5-239	GW223	Sung Yod Pattalung	10	8	80
232	WS21-S5-240	GW084	Chiang Pattalung	10	10	100
233	WS21-S5-241	GW100	Luang Pratiw 123	10	10	100
234	WS21-S5-242	GW179	Tapao Kaew 161	10	6	60
235	WS21-S5-243	GW117	RD19	10	6	60
236	WS21-S5-244	GW159	Kao Banna 432	10	10	100
237	WS21-S5-245	GW157	Kao Tahang 17	10	9	90
238	WS21-S5-246	GW160	Kao Banpong	10	10	100
239	WS21-S5-247	GW051	Kauk Kyi Taung Pyan	10	10	100
240	WS21-S5-248	GW238	Hon Chan	10	10	100
241	WS21-S5-249	DP028	Nakwan	10	10	100
242	WS21-S5-250	GW087	Sedtee	10	10	100
243	WS21-S5-251	W00013	Pawsanhmwe(PSM)	10	7	70
244	WS21-S5-252	GW076	Kao Ruang 88	10	10	100
245	WS21-S5-253	GW182	Nang Kiew	10	9	90
246	WS21-S5-254	GW167	Champa Jean	10	8	80
247	WS21-S5-255	GW143	KonKaew	10	10	100
248	WS21-S5-256	GW010	Chempan	10	10	100
249	WS21-S5-257	GW253	Kao Tahang	10	10	100
250	WS21-S5-258	GW258	Pinkaew	10	6	60
251	WS21-S5-259		Hom Kradung Nga	10	10	100
252	WS21-S5-260	GW234	Lueng Pratan	10	10	100
253	WS21-S5-261	GW259	Puang Tong	10	6	60
254	WS21-S5-262	GW238	Hom Chan	10	10	100
255	WS21-S5-263	GW161	Kao Bunma	10	9	90
256	WS21-S5-264	GW205	Puang Tong 2	10	4	40
257	WS21-S5-265	DP029	Pratan Banbung	10	5	50
258	WS21-S5-266	GW220	Lugdang Pattani	10	10	100
259	WS21-S5-267	DP043	Lueng Kwaylar	10	8	80
260	WS21-S5-268	GW249	Ayuttaya 1	10	10	100
261	WS21-S5-269	GW143	KonKaew 2	10	10	100
262	WS21-S5-270	DP006	Konjud	10	10	100
263	WS21-S5-271	GW175	Dor Kao	10	10	100
264	WS21-S5-272	GW077	Khem	10	10	100
265	WS21-S5-273	GW178	Dee See	10	10	100
266	WS21-S5-274	GW218	Yee Tae Sarming	10	9	90
267	WS21-S5-276	GW200	PinKaew 56	10	10	100
268	WS21-S5-277	GW194	Prachin Buri 2	10	10	100
269	WS21-S5-278	GW088	Sian Oen	10	10	100

Item	seed code	GWAS code	Name	Nubmer of seed test	Number of germinate seed	% Germination
270	WS21-S5-279	DP013	Judmon	10	0	0
271	WS21-S5-280	GW068	PTB33	10	10	100
272	WS21-S5-281	GW089	Niaw Kiaw Ngoo	10	10	100
273	WS21-S5-283	GW208	Phan Tong	10	10	100
274	WS21-S5-284	GW193	Prachin Buri 1	10	3	30
275	WS21-S5-285	W00012	Rathu heenati	10	10	100
276	WS21-S5-286	GW246	Huntra 60	10	8	80
277	WS21-S5-287	GW219	Look Lueng	10	9	90
278	WS21-S5-288	GW113	RD13	10	10	100
279	WS21-S5-289	GW107	Kai Mod Rin 3	10	9	90
280	WS21-S5-290	GW078	Kiew	10	10	100
281	WS21-S5-291	GW169	Chor Lung 97	10	10	100
282	WS21-S5-292	GW255	Chor Maipai	10	7	70
283	NA	W00607	JHN	10	10	100
284	NA	W00608	JHN DH	10	8	80
285	NA	W00610	Nipponbare	10	10	100
286	NA	W00612	PSM	10	9	90
287	NA	W00611	RD6	10	8	80
288	NA	W00613	Lebnok Pattani 2	10	8	80
289	NA		Kao Pongkai 2	10	10	100

Supplementary Table S2 A list of 110 *indica* rice accessions and their callus induction percentage in three different media used for GWAS analysis

Name	N6	SD	B5	SD	MS	SD
Niaw Ubon 1	60.00	23.09	77.78	10.18	86.67	13.33
Khaew	22.22	3.85	64.44	3.85	66.67	6.67
Chumphae 60	60.00	13.33	62.22	10.18	73.33	0.00
KDML105-Bph3-1	75.56	10.18	56.67	4.71	53.33	11.55
RD4	55.56	10.18	56.67	23.57	51.11	23.41
JHN	91.11	3.85	55.56	10.18	53.33	6.67
Leuang Thong	64.44	15.40	55.56	20.37	73.33	11.55
Khao Ban Pong	26.67	24.04	53.33	20.00	26.67	24.04
Phan Thong	60.00	11.55	51.11	7.70	53.33	17.64
MeeKauk	62.22	3.85	46.67	17.64	80.00	13.33
Hom Chan	33.33	6.67	46.67	6.67	6.67	0.00
PTB33	68.89	10.18	44.44	20.37	24.44	7.70
Luang Prathan	22.22	3.85	44.44	7.70	55.56	7.70
RD47	40.00	11.55	44.44	3.85	60.00	13.33
RD6	35.56	10.18	42.22	3.85	44.44	10.18
Setthi	33.33	11.55	40.00	13.33	11.11	3.85
IR62266	56.67	4.71	40.00	0.00	48.89	19.25
Khao Pong Krai	53.33	20.00	40.00	6.67	53.33	11.55
Niew Khiaw Ngoo	46.67	6.67	40.00	11.55	71.11	3.85
Suphan Buri 2	33.33	11.55	40.00	0.00	28.89	16.78
Pokkali	26.67	6.67	40.00	6.67	28.89	13.88
Sang Yod	22.22	10.18	40.00	20.00	57.78	7.70
BENARA	11.11	10.18	40.00	13.33	33.33	6.67
HomMali803-2	62.22	7.70	37.78	3.85	50.00	4.71
Leuang Yai	33.33	6.67	37.78	13.88	44.44	16.78
Pin Kaset 3	28.89	21.43	37.78	16.78	35.56	3.85
Suphan Buri 1	53.33	11.55	37.78	15.40	57.78	7.70
Kalubala Vee	46.67	11.55	37.78	15.40	55.56	7.70

Name	N6	SD	B5	SD	MS	SD
PSM	62.22	3.85	35.56	3.85	28.89	16.78
Pin Kaset 4	35.56	10.18	35.56	3.85	44.44	13.88
DULAR	15.56	13.88	35.56	7.70	60.00	11.55
Leuang Dong	8.89	7.70	35.56	10.18	28.89	3.85
Phuma Dum	28.89	7.70	35.56	13.88	37.78	13.88
Hom Phu Khaew	23.33	4.71	35.56	10.18	20.00	0.00
T6-6	33.33	11.55	33.33	6.67	37.78	10.18
KD20	28.89	20.37	33.33	24.04	68.89	7.70
Dee See	17.78	7.70	33.33	11.55	42.22	16.78
Mae Lad	22.22	23.41	31.11	3.85	22.22	19.25
Leuang Patew 123	42.22	16.78	31.11	13.88	11.11	7.70
CNTLR82006-KSR-2-7	35.56	10.18	28.89	10.18	28.89	3.85
Prachin Buri 2	33.33	11.55	28.89	3.85	0.00	0.00
Phuang Nug	22.22	10.18	28.89	3.85	20.00	6.67
Dor Khao	15.56	7.70	28.89	15.40	37.78	13.88
Hom Phama	8.89	7.70	28.89	15.40	28.89	10.18
Phitsanulok 60-1	84.44	3.85	26.67	6.67	37.78	3.85
BA7	40.00	11.55	26.67	6.67	46.67	0.00
Khao Khum	31.11	7.70	26.67	13.33	35.56	16.78
Sri Nuan	26.67	13.33	26.67	6.67	24.44	13.88
Rathu heenati	24.44	3.85	26.67	6.67	24.44	10.18
DH103	22.22	3.85	26.67	6.67	44.44	13.88
Rice Berry	20.00	20.00	26.67	11.55	37.78	10.18
Chiang Phatthalung	0.00	0.00	26.67	6.67	33.33	6.67
Hom Chan	37.78	3.85	24.44	7.70	35.56	3.85
RD27	33.33	20.00	24.44	15.40	33.33	0.00
Khao Klang	17.78	7.70	24.44	13.88	20.00	6.67
Chempan	22.22	3.85	24.44	10.18	33.33	6.67
Kon Kaew	20.00	6.67	24.44	10.18	24.44	7.70
Pin Kaew 56	2.22	3.85	24.44	10.18	40.00	23.09
Khao Kor Diaw 35	33.33	19.25	22.22	16.78	31.11	10.18

Name	N6	SD	B5	SD	MS	SD
Num SaKui 19	17.78	7.70	22.22	7.70	22.22	3.85
Hom Khlong Luang 1	44.44	7.70	20.00	20.00	35.56	13.88
Leuang Kamin	30.00	4.71	20.00	9.43	48.89	3.85
Hom Dong	24.44	3.85	20.00	0.00	35.56	3.85
Hom Satabhutra	24.44	3.85	20.00	13.33	22.22	21.43
Khao Banna 432	37.78	7.70	17.78	7.70	57.78	16.78
Lug Dang Pattani	33.33	13.33	17.78	3.85	46.67	17.64
TDK1-Sub	31.11	23.41	17.78	3.85	24.44	3.85
Khao Ta Hang	26.67	6.67	17.78	7.70	31.11	3.85
Hom Lao	20.00	13.33	17.78	7.70	26.67	6.67
Khao Ruang 88	15.56	13.88	17.78	20.37	17.78	7.70
Chor Lung 97	8.89	3.85	17.78	3.85	2.22	3.85
IR40	2.22	3.85	17.78	3.85	4.44	7.70
Niaw Sonpatong	28.89	10.18	16.67	4.71	15.56	3.85
Hom Nang Nuan	46.67	0.00	15.56	7.70	48.89	10.18
Nakwan	22.22	3.85	15.56	13.88	53.33	17.64
Konjood	6.67	6.67	15.56	10.18	28.89	15.40
Mali Dang	28.89	10.18	13.33	0.00	35.56	10.18
Kon Kaew	28.89	10.18	13.33	6.67	6.67	0.00
RD13	26.67	17.64	13.33	6.67	40.00	6.67
HomMali803-1	24.44	16.78	13.33	7.70	73.33	6.67
Sian Orn	20.00	11.55	13.33	6.67	11.11	10.18
CNTBR82040-259-1-1-1	17.78	3.85	13.33	9.43	33.33	18.86
Taban	17.78	3.85	13.33	6.67	20.00	6.67
Surin 1	16.67	4.71	13.33	6.67	6.67	11.55
MNTK75	13.33	11.55	13.33	0.00	35.56	7.70
Mark Num	6.67	6.67	13.33	11.55	22.22	13.88
Ruang Diaw	23.33	4.71	11.11	10.18	24.44	10.18
E-pid	23.33	4.71	11.11	7.70	20.00	11.55
Hom Thong	17.78	15.40	11.11	3.85	48.89	13.88
Niaw Dum	17.78	13.88	11.11	3.85	26.67	6.67

Name	N6	SD	B5	SD	MS	SD
RD8	13.33	6.67	11.11	10.18	17.78	16.78
Khao Nok	8.89	7.70	11.11	3.85	6.67	6.67
Khao Ngachang	37.78	15.40	8.89	7.70	24.44	13.88
Mabang	33.33	13.33	8.89	10.18	33.33	13.33
Ayutthaya 1	22.22	3.85	8.89	3.85	28.89	13.88
Nipponbare	13.33	6.67	8.89	3.85	20.00	6.67
Char Makok	8.89	7.70	8.89	3.85	22.22	21.43
RD37	37.78	19.25	6.67	11.55	42.22	7.70
Rak Hang	16.67	4.71	6.67	9.43	24.44	3.85
RD7	8.89	10.18	4.44	3.85	33.33	24.04
RD11	6.67	6.67	4.44	3.85	15.56	7.70
Tom Muangluang	0.00	0.00	4.44	7.70	0.00	0.00
Phitsanulok 80	15.56	10.18	3.33	4.71	3.33	4.71
Kemthong Phatthalung	35.56	7.70	2.22	3.85	24.44	7.70
Thong Raksai	17.78	3.85	2.22	3.85	22.22	16.78
Kee Thom Klang	4.44	7.70	2.22	3.85	2.22	3.85
RD39	0.00	0.00	2.22	3.85	6.67	6.67
Suphan Buri 4	6.67	9.43	0.00	0.00	10.00	4.71
Jao Hom Suphan Buri	4.44	7.70	0.00	0.00	26.67	9.43
Suphan Buri 90	4.44	7.70	0.00	0.00	4.44	7.70
Kan Chan	0.00	0.00	0.00	0.00	0.00	0.00

Supplementary Table S3 A list of candidate genes for the callus induction in the qCI-B5-Chr6; the golden highlight(s) showed the gene(s) located at the significant SNP of the QTL.

Gene ID	GenePosStart	GenePosEnd	Description
Os06g0253350	7954357	7958130	Hypothetical gene. (Os06t0253350-00)
Os06g0253600	7964478	7967674	Similar to secretory protein-like. (Os06t0253600-00)
Os06g0254200	7982027	7983859	Similar to Potassium channel protein NKT5. (Os06t0254200-00)
Os06g0254300	7983970	7990956	Similar to EF-hand calcium bining protein. (Os06t0254300-01)
Os06g0254600	7996917	8004842	Caleosin related family protein. (Os06t0254600-01)
Os06g0254700	8011879	8014834	Caleosin related family protein. (Os06t0254700-01)
Os06g0254901	8029753	8030718	Non-protein coding transcript. (Os06t0254901-00)
Os06g0255001	8034209	8035027	Non-protein coding transcript. (Os06t0255001-00)
Os06g0255100	8038476	8041831	2OG-Fe(II) oxygenase domain containing protein. (Os06t0255100-01)
Os06g0255200	8043566	8067365	Conserved hypothetical protein. (Os06t0255200-01)
Os06g0255400	8069204	8069983	Similar to Nudix hydrolase 18, mitochondrial precursor (EC 3.6.1.-) (AtNUDT18). (Os06t0255400-01)
Os06g0255700	8097302	8104472	DEAD-like helicase, N-terminal domain containing protein. (Os06t0255700-01)
Os06g0255900	8116249	8119845	Conserved hypothetical protein. (Os06t0255900-01)
Os06g0256000	8127437	8129334	Conserved hypothetical protein. (Os06t0256000-01)
Os06g0256200	8129560	8134453	Similar to Keratinocytes proline-rich protein. (Os06t0256200-01)
Os06g0256300	8142728	8147085	Similar to Calmodulin-binding heat-shock protein. (Os06t0256300-01)
Os06g0256500	8152444	8159313	Similar to Glucose-6-phosphate isomerase. (Os06t0256500-01)
Os06g0256600	8162028	8166874	Conserved hypothetical protein. (Os06t0256600-01)
Os06g0256800	8176855	8177675	Hypothetical conserved gene. (Os06t0256800-01)
Os06g0256900	8178991	8184548	Glycoside hydrolase family 9 subclass B3, Endo-beta-1,4-glucanase, Lignocellulose crystallinity modification in stem internode growth and development, Plant strength, cellulose modification, and biomass saccharification (Os06t0256900-01)
Os06g0257050	8179502	8184497	Hypothetical protein. (Os06t0257050-00)
Os06g0257200	8194555	8198058	Similar to Signal recognition particle 9 kDa protein. (Os06t0257200-01)
Os06g0257450	8230555	8232480	Ribonucleotide reductase, Chloroplast biogenesis (Os06t0257450-01)
Os06g0257600	8236902	8238699	Esterase, SGNH hydrolase-type domain containing protein. (Os06t0257600-01)
Os06g0258000	8251038	8252390	Similar to Typical P-type R2R3 Myb protein (Fragment). (Os06t0258000-01)
Os06g0258500	8283058	8283486	Myb transcription factor domain containing protein. (Os06t0258500-00)
Os06g0258900	8301780	8316896	NAD(P)-binding domain containing protein. (Os06t0258900-01)
Os06g0259000	8319050	8321692	Hypothetical conserved gene. (Os06t0259000-01)

Supplementary Table S4 A list of candidate genes for the callus induction in the qCI-MS-Chr2; the golden highlight(s) showed the gene(s) located at the significant SNP of the QTL.

Gene ID	GenePosStart	GenePosEnd	Description
Os02g0255900	8825029	8825614	Conserved hypothetical protein. (Os02t0255900-01)
Os02g0256000	8834020	8835881	Hypothetical protein. (Os02t0256000-01)
Os02g0256100	8834369	8837492	Pectin lyase fold/virulence factor domain containing protein. (Os02t0256100-01)
Os02g0256200	8842948	8844249	Histidine- and alanine-rich protein, Leaf sheath elongation (Os02t0256200-02)
Os02g0256500	8850623	8851897	Conserved hypothetical protein. (Os02t0256500-01)
Os02g0256800	8854327	8854985	Cupredoxin domain containing protein. (Os02t0256800-00)
Os02g0257001	8856566	8860576	Hypothetical conserved gene. (Os02t0257001-00)
Os02g0257100	8866815	8867667	Cupredoxin domain containing protein. (Os02t0257100-01)
Os02g0257200	8869192	8871520	Conserved hypothetical protein. (Os02t0257200-01)
Os02g0257250	8869357	8871474	Hypothetical gene. (Os02t0257250-00)
Os02g0257300	8873101	8875996	Rhodanese-like domain containing protein. (Os02t0257300-00)
Os02g0257400	8876190	8876441	Non-protein coding transcript. (Os02t0257400-01)
Os02g0257500	8879339	8882660	Basic helix-loop-helix (bHLH) transcription factor, Initiation of stomatal development, Control of stomatal initiation and meristemoid fate (Os02t0257500-01)
Os02g0258000	8927818	8928740	Hypothetical conserved gene. (Os02t0258000-00)
Os02g0258200	8935193	8936223	Similar to high mobility group family. (Os02t0258200-01)
Os02g0258250	8936361	8937179	Hypothetical conserved gene. (Os02t0258250-00)
Os02g0258300	8938133	8943482	Zinc finger, FYVE/PHD-type domain containing protein. (Os02t0258300-01)
Os02g0258800	8973633	8977322	Conserved hypothetical protein. (Os02t0258800-01)
Os02g0258900	8976792	8981140	Similar to Molybdopterin biosynthesis CNX2 protein (Molybdenum cofactor biosynthesis enzyme CNX2). (Os02t0258900-01)
Os02g0259100	8984621	8989570	Conserved hypothetical protein. (Os02t0259100-01)
Os02g0259600	9005264	9007486	Similar to 50S ribosomal protein L21, chloroplast precursor (CL21) (CS-L7). (Os02t0259600-01)
Os02g0259850	9016923	9018833	Hypothetical gene. (Os02t0259850-00)
Os02g0259900	9021454	9023102	Conserved hypothetical protein. (Os02t0259900-01)
Os02g0260000	9026460	9026900	Non-protein coding transcript. (Os02t0260000-01)
Os02g0260200	9042064	9046144	Kelch repeat-containing F-box protein, Regulation of panicle architecture (Os02t0260200-01)
Os02g0260400	9052733	9056389	Protein of unknown function DUF37 family protein. (Os02t0260400-01)
Os02g0260500	9066936	9068429	Hypothetical conserved gene. (Os02t0260500-00)
Os02g0260700	9082763	9087989	Similar to GAMYB-binding protein (Fragment). (Os02t0260700-01)
Os02g0260800	9091400	9091461	Non-protein coding transcript. (Os02t0260800-00)

Gene ID	GenePosStart	GenePosEnd	Description
Os02g0260900	9095708	9096382	Similar to H0315A08.1 protein. (Os02t0260900-00)

Supplementary Table S5 A list of candidate genes for the callus induction in the qCI-MS-Chr6; the golden highlight(s) showed the gene(s) located at the significant SNP of the QTL.

Gene ID	GenePosStart	GenePosEnd	Description
Os06g0486400	16604884	16616223	Serine/threonine protein kinase domain containing protein. (Os06t0486400-01)
Os06g0486800	16649553	16653244	Similar to Formate dehydrogenase, mitochondrial precursor (EC 1.2.1.2) (NAD- dependent formate dehydrogenase) (FDH). (Os06t0486800-01)
Os06g0486900	16676478	16683024	Similar to Formate dehydrogenase, mitochondrial precursor (EC 1.2.1.2) (NAD- dependent formate dehydrogenase) (FDH). (Os06t0486900-01)
Os06g0487300	16693757	16694391	Hypothetical protein. (Os06t0487300-01)
Os06g0487380	16693769	16694662	Hypothetical gene. (Os06t0487380-01)
Os06g0487620	16705088	16705640	Conserved hypothetical protein. (Os06t0487620-01)
Os06g0487660	16719026	16719110	Non-protein coding transcript. (Os06t0487660-00)
Os06g0487700	16719409	16719801	Hypothetical conserved gene. (Os06t0487700-00)
Os06g0487900	16732927	16745662	SUMO (Small Ubiquitin-like Modifier) Protease, Salt tolerance (Os06t0487900-01)
Os06g0488050	16763476	16768965	Similar to Protein kinase family protein. (Os06t0488050-00)
Os06g0488125	16763594	16768949	Hypothetical protein. (Os06t0488125-00)
Os06g0488200	16774133	16785410	Similar to Myosin heavy chain (Fragment). (Os06t0488200-01)
Os06g0488600	16793652	16795663	Similar to Potential phospholipid-transporting ATPase 7 (EC 3.6.3.1) (Aminophospholipid flippase 7). (Os06t0488600-01)
Os06g0489200	16825601	16836194	Protein of unknown function DUF1604 domain containing protein. (Os06t0489200-01)
Os06g0489500	16852004	16858593	CMP/dCMP deaminase, zinc-binding domain containing protein. (Os06t0489500-01)
Os06g0489900	16877329	16878690	Domain of unknown function DUF1618 domain containing protein. (Os06t0489900-00)
Os06g0490000	16879618	16881668	Similar to DIMETHYLADENOSINE TRANSFERASE. (Os06t0490000-01)
Os06g0490200	16886792	16888459	Conserved hypothetical protein. (Os06t0490200-01)
Os06g0490400	16896478	16897774	Similar to Class III peroxidase 80. (Os06t0490400-00)
Os06g0490700	16910985	16916624	Conserved hypothetical protein. (Os06t0490700-01)
Os06g0491300	16963479	16966760	Hypothetical conserved gene. (Os06t0491300-01)
Os06g0491566	16968153	16970345	Conserved hypothetical protein. (Os06t0491566-01)
Os06g0491800	16991828	16994085	HAT dimerisation domain containing protein. (Os06t0491800-01)
Os06g0491901	16998507	17002390	Non-protein coding transcript. (Os06t0491901-01)
Os06g0492000	17003636	17010230	Similar to Phosphatidylinositol synthase. (Os06t0492000-01)
Os06g0492101	17006585	17008463	Hypothetical conserved gene. (Os06t0492101-00)

Supplementary Table S6 A list of candidate genes for the callus induction in the qCI-N6-Chr6.1; the golden highlight(s) showed the gene(s) located at the significant SNP of the QTL.

Gene ID	GenePosStart	GenePosEnd	Description
Os06g0165500	3305304	3307872	S-Domain receptor like kinase-37, Response to drought in tolerant genotypes (Os06t0165500-00)
Os06g0165600	3310919	3311822	Homolog of CBF/DREB1 (Os06t0165600-01)
Os06g0165800	3314114	3315452	Similar to Caffeoyl-CoA 3-O-methyltransferase (Fragment). (Os06t0165800-01)
Os06g0165900	3316737	3317378	Conserved hypothetical protein. (Os06t0165900-00)
Os06g0166000	3319112	3322249	Cyclin-like F-box domain containing protein. (Os06t0166000-01)
Os06g0166050	3321838	3322412	Hypothetical gene. (Os06t0166050-00)
Os06g0166100	3322909	3327600	FAR1 domain containing protein. (Os06t0166100-01)
Os06g0166200	3328557	3331513	Zinc finger, C2H2-type domain containing protein. (Os06t0166200-01)
Os06g0166400	3337151	3338155	Similar to TINY-like protein (AP2 domain containing protein RAP2.10) (Fragment). (Os06t0166400-01)
Os06g0166500	3342464	3346318	Aux/IAA protein, Mediation of abiotic stress tolerance, Drought and salt tolerance (Os06t0166500-01)
Os06g0166900	3360524	3363523	Protein kinase, core domain containing protein. (Os06t0166900-01)
Os06g0167000	3363900	3371061	Similar to predicted protein. (Os06t0167000-01)
Os06g0167100	3376576	3386418	Armadillo-like helical domain containing protein. (Os06t0167100-01)
Os06g0167125	3385199	3386449	Non-protein coding transcript. (Os06t0167125-00)
Os06g0167150	3388823	3390644	Hypothetical gene. (Os06t0167150-01)
Os06g0167200	3389074	3390445	Similar to RING-H2 finger protein ATL1R (RING-H2 finger protein ATL8). (Os06t0167200-01)
Os06g0167400	3405405	3407716	Di-trans-poly-cis-decaprenylcistransferase family protein. (Os06t0167400-01)
Os06g0167500	3408660	3418587	Leucine-rich repeat, plant specific containing protein. (Os06t0167500-01)
Os06g0167600	3420507	3421462	Similar to Proteasome subunit alpha-3 (Fragment). (Os06t0167600-01)
Os06g0168000	3428704	3433378	Glutathione S-transferase, C-terminal-like domain containing protein. (Os06t0168000-01)
Os06g0168150	3431958	3432897	Hypothetical gene. (Os06t0168150-00)
Os06g0168400	3440104	3443075	RNA polymerase Rpb7, N-terminal domain containing protein. (Os06t0168400-01)
Os06g0168500	3445034	3448312	Qa-SNARE (soluble N-ethylmaleimide sensitive factor attachment protein receptor), t-SNARE, Regulation of arbuscular mycorrhizal (AM) symbiosis (Os06t0168500-01)
Os06g0168600	3449701	3454747	Ribonucleotide reductase, Chloroplast biogenesis (Os06t0168600-01)
Os06g0168700	3455800	3456811	Similar to Prolin rich protein. (Os06t0168700-01)
Os06g0168800	3460493	3465373	Similar to Protein kinase. (Os06t0168800-01)
Os06g0168901	3468467	3469940	Hypothetical conserved gene. (Os06t0168901-00)

Gene ID	GenePosStart	GenePosEnd	Description
Os06g0169001	3474629	3476370	GOS9 protein. (Os06t0169001-01)
Os06g0169600	3490289	3490874	Similar to Beta-tubulin (Fragment). (Os06t0169600-00)
Os06g0169800	3504741	3505001	Hypothetical protein. (Os06t0169800-00)
Os06g0169900	3507359	3509141	Similar to GOS9 protein. (Os06t0169900-00)
Os06g0170000	3513202	3514396	Non-protein coding transcript. (Os06t0170000-00)
Os06g0170100	3515130	3518031	Similar to WAK80 - OsWAK receptor-like protein kinase. (Os06t0170100-00)
Os06g0170200	3519052	3520463	Conserved hypothetical protein. (Os06t0170200-00)
Os06g0170500	3536683	3540219	Similar to RNA-binding protein-like. (Os06t0170500-01)
Os06g0170800	3558158	3559922	Similar to H0124B04.16 protein. (Os06t0170800-01)
Os06g0170866	3559947	3561401	F-box domain, cyclin-like domain containing protein. (Os06t0170866-00)
Os06g0171500	3585217	3588270	Conserved hypothetical protein. (Os06t0171500-01)
Os06g0171600	3588217	3595865	Membrane insertion protein, OxaA/YidC domain containing protein. (Os06t0171600-01)
Os06g0171700	3596001	3598291	Cdk-activating kinase assembly factor (MAT1) family protein. (Os06t0171700-01)
Os06g0171800	3598466	3605188	VRR-NUC domain containing protein. (Os06t0171800-01)
Os06g0171900	3632670	3639453	WD40 subfamily protein, Salt stress (Os06t0171900-01)
Os06g0172000	3640786	3643222	Pentatricopeptide repeat domain containing protein. (Os06t0172000-01)
Os06g0172101	3649379	3650265	Hypothetical gene. (Os06t0172101-00)
Os06g0172200	3649517	3650686	EF-Hand type domain containing protein. (Os06t0172200-02)
Os06g0172600	3656494	3657055	Similar to predicted protein. (Os06t0172600-00)
Os06g0172675	3660715	3661175	Hypothetical protein. (Os06t0172675-00)
Os06g0172750	3663062	3664355	Non-protein coding transcript. (Os06t0172750-01)
Os06g0172800	3675795	3679085	Similar to alkaline alpha galactosidase 2. (Os06t0172800-00)
Os06g0172901	3676277	3678331	Hypothetical protein. (Os06t0172901-00)
Os06g0173000	3682377	3688555	Armadillo-type fold domain containing protein. (Os06t0173000-00)
Os06g0173100	3689676	3694342	26S proteasome 19S regulatory particle triple-A ATPase subunit 5b (Os06t0173100-01)
Os06g0173450	3692016	3693281	Hypothetical protein. (Os06t0173450-00)

Supplementary Table S7 A list of candidate genes for the callus induction in the qCI-N6-Chr6.2; the golden highlight(s) showed the gene(s) located at the significant SNP of the QTL.

**Gene ID**	**GenePosStart**	**GenePosEnd**	**Description**
Os06g0250600	7799085	7806567	Outward Shaker K⁺ channel, K⁺ release by guard cells for stomatal closure, K⁺ translocation toward the shoots (Os06t0250600-01)
Os06g0251000	7825468	7829696	Similar to mitochondrion protein. (Os06t0251000-01)
Os06g0251100	7835332	7840950	Membrane attack complex component/perforin (MACPF) domain domain containing protein. (Os06t0251100-00)
Os06g0251200	7841699	7844795	Kelch related domain containing protein. (Os06t0251200-01)
Os06g0251700	7848384	7854067	Myosin II heavy chain-like family protein. (Os06t0251700-01)
Os06g0251900	7848697	7854050	Hypothetical protein. (Os06t0251900-00)
Os06g0252300	7920702	7923482	Hypothetical conserved gene. (Os06t0252300-01)
Os06g0252800	7933984	7935241	Similar to STF-1 (Fragment). (Os06t0252800-01)
Os06g0253100	7940956	7941680	Heat shock protein Hsp20 domain containing protein. (Os06t0253100-01)
Os06g0253350	7954357	7958130	Hypothetical gene. (Os06t0253350-00)
Os06g0253600	7964478	7967674	Similar to secretory protein-like. (Os06t0253600-00)
Os06g0254200	7982027	7983859	Similar to Potassium channel protein NKT5. (Os06t0254200-00)
Os06g0254300	7983970	7990956	Similar to EF-hand calcium bining protein. (Os06t0254300-01)
Os06g0254600	7996917	8004842	Caleosin related family protein. (Os06t0254600-01)
Os06g0254700	8011879	8014834	Caleosin related family protein. (Os06t0254700-01)
Os06g0254901	8029753	8030718	Non-protein coding transcript. (Os06t0254901-00)
Os06g0255001	8034209	8035027	Non-protein coding transcript. (Os06t0255001-00)
Os06g0255100	8038476	8041831	2OG-Fe(II) oxygenase domain containing protein. (Os06t0255100-01)
Os06g0255200	8043566	8067365	Conserved hypothetical protein. (Os06t0255200-01)
Os06g0255400	8069204	8069983	Similar to Nudix hydrolase 18, mitochondrial precursor (EC 3.6.1.-) (AtNUDT18). (Os06t0255400-01)
Os06g0255700	8097302	8104472	DEAD-like helicase, N-terminal domain containing protein. (Os06t0255700-01)
Os06g0255900	8116249	8119845	Conserved hypothetical protein. (Os06t0255900-01)
Os06g0256000	8127437	8129334	Conserved hypothetical protein. (Os06t0256000-01)
Os06g0256200	8129560	8134453	Similar to Keratinocytes proline-rich protein. (Os06t0256200-01)
Os06g0256300	8142728	8147085	Similar to Calmodulin-binding heat-shock protein. (Os06t0256300-01)
Os06g0256500	8152444	8159313	Similar to Glucose-6-phosphate isomerase. (Os06t0256500-01)
Os06g0256600	8162028	8166874	Conserved hypothetical protein. (Os06t0256600-01)
Os06g0256800	8176855	8177675	Hypothetical conserved gene. (Os06t0256800-01)
Os06g0256900	8178991	8184548	Glycoside hydrolase family 9 subclass B3, Endo-beta-1,4-glucanase, Lignocellulose crystallinity modification in stem internode growth and development, Plant strength, cellulose modification, and biomass saccharification (Os06t0256900-01)
Os06g0257050	8179502	8184497	Hypothetical protein. (Os06t0257050-00)
Os06g0257200	8194555	8198058	Similar to Signal recognition particle 9 kDa protein. (Os06t0257200-01)

Supplementary Table S8 A list of candidate genes for the callus induction in the qCI-N6-Chr7; the golden highlight(s) showed the gene(s) located at the significant SNP of the QTL.

Gene ID	GenePosStart	GenePosEnd	Description
Os07g0255200	8704268	8706000	Similar to Helicase-like protein [Oryza sativa (japonica cultivar-group)]. (Os07t0255200-01)
Os07g0255300	8709616	8710266	Conserved hypothetical protein. (Os07t0255300-01)
Os07g0255900	8783519	8785197	Domain of unknown function DUF231, plant domain containing protein. (Os07t0255900-00)
Os07g0256200	8807273	8811445	RNA recognition motif, RNP-1 domain containing protein. (Os07t0256200-01)
Os07g0256300	8812366	8815314	Conserved hypothetical protein. (Os07t0256300-00)
Os07g0256700	8828926	8830964	Protein of unknown function DUF231, plant domain containing protein. (Os07t0256700-01)
Os07g0256866	8845266	8857772	Hypothetical gene. (Os07t0256866-00)

Supplementary Table S9 A list of candidate genes for the callus induction in the qCI-N6-Chr11; the golden highlight(s) showed the gene(s) located at the significant SNP of the QTL.

Gene ID	GenePosStart	GenePosEnd	Description
Os11g0636600	25134253	25137270	Conserved hypothetical protein. (Os11t0636600-01)
Os11g0636900	25148722	25153451	Similar to Splicing factor U2af large subunit A. (Os11t0636900-01)
Os11g0637000	25153799	25155522	Similar to Sugar transporter family protein. (Os11t0637000-01)
Os11g0637050	25153911	25155463	Hypothetical protein. (Os11t0637050-00)
Os11g0637100	25160019	25161761	Similar to Sugar transporter family protein. (Os11t0637100-00)
Os11g0637166	25162871	25164608	Hypothetical gene. (Os11t0637166-01)
Os11g0637200	25165988	25167744	Similar to Sorbitol transporter. (Os11t0637200-01)
Os11g0637300	25171719	25172081	Hypothetical conserved gene. (Os11t0637300-00)
Os11g0637501	25177818	25180272	Similar to membrane protein. (Os11t0637501-00)
Os11g0637600	25182326	25183324	Similar to potyvirus VPg interacting protein. (Os11t0637600-00)
Os11g0637700	25193013	25197347	RNA-binding protein, Heterogeneous nuclear ribonucleoprotein 1 (hnRNP1), Regulation of spikelet hull length (Os11t0637700-01)
Os11g0637800	25199882	25206038	Similar to 1-acyl-sn-glycerol-3-phosphate acyltransferase PLS1. (Os11t0637800-01)
Os11g0637900	25205090	25206092	Hypothetical gene. (Os11t0637900-00)
Os11g0638000	25213451	25216758	Similar to GTP-binding protein engA. (Os11t0638000-01)
Os11g0638200	25218327	25222357	Conserved hypothetical protein. (Os11t0638200-00)
Os11g0638700	25237345	25239585	Protein of unknown function DUF594 domain containing protein. (Os11t0638700-00)
Os11g0638802	25238381	25239594	Non-protein coding transcript. (Os11t0638802-00)
